# Hot water extract of *Agaricus blazei* Murrill specifically inhibits growth and induces apoptosis in human pancreatic cancer cells

**DOI:** 10.1186/s12906-018-2385-4

**Published:** 2018-12-04

**Authors:** Yoshihisa Matsushita, Yoshiyuki Furutani, Rumiko Matsuoka, Toru Furukawa

**Affiliations:** 1 International Research and Educational Institute for Integrated Medical Sciences and Integrated Medical Sciences, Advanced Biomedical Engineering and Science, Graduate School of Medicine, Tokyo Women’s Medical University, 8-1 Kawadacho, Shinjuku, Tokyo, 162-8666 Japan; 2 International Research and Educational Institute for Integrated Medical Sciences and Division of Pediatric Cardiology, Tokyo Women’s Medical University, 8-1 Kawadacho, Shinjuku, Tokyo, 162-8666 Japan; 3 Wakamatsu-Kawada Clinic, 10-7 Kawadacho, Shinjuku, Tokyo, 162-0054 Japan; 4 International Research and Educational Institute for Integrated Medical Sciences and Institute for Integrated Medical Sciences, Tokyo Women’s Medical University, 8-1 Kawadacho, Shinjuku, Tokyo, 162-8666 Japan

**Keywords:** Pancreatic cancer, Drug development, *Agaricus blazei* Murrill, Apoptosis, Gene expression, Kinetochore, Spindle, Cell cycle

## Abstract

**Background:**

Pancreatic cancer is one of the most aggressive human malignancies. The development of a novel drug to treat pancreatic cancer is imperative, and it is thought that complementary and alternative medicine (CAM) could yield such a candidate. *Agaricus blazei* Murrill is a CAM that has been tested as an anticancer drug, but its efficacy against pancreatic cancer is poorly understood. To study the potential of *A. blazei* in the treatment of pancreatic cancer, we examined the effects of its hot water extract on the proliferation and global gene expression profile of human pancreatic cancer cells.

**Methods:**

Three distinct human pancreatic cancer cell lines, MIAPaCa-2, PCI-35, and PK-8, and the immortalized human pancreatic duct-epithelial cell line, HPDE, were employed. The cells were incubated with the appropriate growth medium supplemented with the hot water extract of *A. blazei* at final concentrations of 0.005, 0.015%, or 0.045%, and cellular proliferation was assessed for five consecutive days using an MTT assay. Apoptosis was examined by using flow cytometry and the terminal deoxynucleotidyl transferase dUTP nick-end labeling (TUNEL) assay. Caspase-dependent apoptosis was assayed using immunoblotting. Global gene expression profiles were examined using a whole human genome 44 K microarray, and the microarray results were validated by using real-time reverse transcription PCR.

**Results:**

The hot water extract of *A. blazei* significantly inhibited the proliferation of cultured pancreatic cancer cells through the induction of G0/G1 cell cycle arrest and caspase-dependent apoptosis; the effect was the smallest in HPDE cells. Furthermore, significant alterations in the global gene expression profiles of pancreatic cancer cells occurred following treatment with the hot water extract of *A. blazei*. Genes associated with kinetochore function, spindle formation, and centromere maintenance were particularly affected, as well as cyclins and cyclin-dependent kinases that are essential for cell cycle progression. In addition, proapoptotic genes were upregulated.

**Conclusions:**

The hot water extract of *A. blazei* may be useful for the treatment of pancreatic cancer and is a potential candidate for the isolation of novel, active compounds specific for mitotic spindle dysfunction.

**Electronic supplementary material:**

The online version of this article (10.1186/s12906-018-2385-4) contains supplementary material, which is available to authorized users.

## Background

In 2012, pancreatic cancer was estimated to be the twelfth most common cancer in men and the eleventh most common in women, with an estimated 330,000 deaths occurring worldwide. Because of this very high mortality rate, comprising an overall mortality-to-incidence ratio of 0.98 and a 5-year survival rate of 5%, pancreatic cancer ranks as the seventh most common cause of death from cancer worldwide. The leading identified cause of pancreatic cancer is cigarette smoking, but other risk factors include older age, race, obesity, diabetes mellitus, and chronic pancreatitis [1]. The signs and symptoms of pancreatic cancer include back pain, unexplained weight loss, jaundice, and pruritus. New-onset diabetes can be the first manifestation of pancreatic cancer [2]. The most effective therapeutic option for pancreatic cancer is a curative surgical resection with adjuvant chemotherapy; however, only 15–20% of pancreatic cancer patients are candidates for surgery/chemotherapy. After this curative operation, the 5-year survival rate approaches 20% [3].

Gemcitabine has been a systemic first-line chemotherapeutic drug for pancreatic cancer patients for nearly two decades; however, it has limited efficacy and the mean survival period is only 6 months [4]. Recently, two randomized controlled trials showed a moderate advantage of combination therapies over gemcitabine: FOLFIRINOX (folinic acid, 5-fluorouracil, irinotecan, and oxaliplatin) prolonged patient survival to 11.1 months (vs. 6.8 months with gemcitabine alone) [5], and gemcitabine plus nab-paclitaxel prolonged survival to 8.5 months (vs. 6.7 months with gemcitabine alone) [6]. However, most patients experienced relapses, although they managed to tolerate the increased drug toxicity. Therefore, it is imperative to find a novel compound for the treatment of pancreatic cancer that is more effective and has a lower burden of toxicity.

Complementary and alternative medicine (CAM) has been used for the treatment of numerous chronic disease conditions, including cancer [7]. Mushrooms are the most frequently used CAM in Japan, with *Agaricus blazei* Murrill, a medicinal mushroom commonly known as “Himematsutake,” accounting for 60.6% of the medicinal mushroom usage in Japan. *A. blazei* has been tested for the treatment of cancer, viral diseases, and diabetes mellitus [8–13]. We previously found that a hot water extract of *A. blazei* (AbE) could improve cardiovascular symptoms in patients; subsequently, we found that brefeldin A, a compound purified from AbE, had estrogenic activity [14, 15]. Experiments have confirmed the ability of AbE to inhibit the growth of various cancers, including colon cancer, leukemia, methylcholanthrene-induced fibrosarcoma, Sarcoma-180, Ehrlich ascites carcinoma, and Shionogi carcinoma [16–19]. AbE was shown to induce apoptosis in cancer cells through the Bax- and the caspase-dependent pathway and to modulate immunological reactions through an increase in the production of tumor necrosis factor-α (TNF-α) and interleukin-8 (IL-8) by macrophages [20–22]. Despite these studies, the detailed mechanistic pathways of these pharmaceutical effects remain unknown. Moreover, despite the current prevalence of genomic investigations in scientific research, the effect of AbE on global gene expression in human cells has not been investigated.

To determine the potential of AbE for the treatment of pancreatic cancer, we compared its effects on the proliferation of human pancreatic cancer cells and normal pancreatic duct-epithelial cells. Moreover, to gain mechanistic insight into the effects of AbE, we explored the global gene expression profiles of pancreatic cancer cells treated with AbE.

## Methods

### Preparation of AbE

*A. blazei* powder (*A. blazei* mycelia-dikaryon, strain my26) was provided by JMCU Center Corporation [14]. The powder (2.5 g) was boiled in 45 mL water for 10 min. The supernatant was recovered by centrifugation at 3300×*g* for 3 min and sterilized by filtration through a 0.22-μm membrane. The solution obtained (AbE) was used for the following experiments.

### Cell culture

Three human pancreatic cancer cell lines, MIA PaCa-2, PCI-35, and PK-8, and the immortalized human pancreatic duct epithelial cell line, HPDE, were used in this study. MIA PaCa-2 was purchased from American Type Culture Collection (Manassas, VA, USA). PCI-35 was obtained from Dr. Hiroshi Ishikura at Department of Pathology, Hokkaido University Graduate School of Medicine, Sapporo, Japan [23]. PK-8 was obtained from Dr. Masao Kobari at the Department of Surgery, Tohoku University Graduate School of Medicine, Sendai, Japan [24]. HPDE was obtained from Dr. Ming-Sound Tsao, Department of Pathology, Princess Margaret Hospital and Toronto University, Toronto, Canada [25]. PCI-35 and PK-8 were cultured in RPMI 1640 (Sigma-Aldrich Corp., St. Louis, MO, USA) supplemented with 10% fetal bovine serum (Biowest, Nuaillé, France). MIA PaCa-2 was cultured in DMEM (Sigma-Aldrich Corp.) supplemented with 10% fetal bovine serum (Biowest). HPDE was maintained in Medium 154 (Cascade Biologics, Portland, OR, USA) containing 1% human keratinocyte growth supplement (Cascade Biologics). All cells were maintained at 37 °C in a humid atmosphere with 5% CO_2_.

### Cell proliferation assay

For this assay, 5000 cells/well were plated in 100 μL of the appropriate culture medium in 32 wells of a 96-well plate; in total, 5 plates were used. The cells were incubated for 24 h and then the culture medium of 8 wells was replaced with medium supplemented with AbE at final concentrations of 0.005, 0.015%, or 0.045% (*w*/*v*). For the control (MOCK), an equal volume of water was added to the medium of the remaining 8 wells. On each assay day, the medium from each of the 32 wells in one plate was replaced with 100 μL of 0.05% 3-(4,5-dimethylthiazol-2-yl)-2,5-diphenyltetrazolium bromide (MTT)/PBS(−), and the plate was then incubated for 1 h at 37 °C in an atmosphere containing 5% CO_2_. After incubation, the MTT solution was removed by aspiration, the cells were resuspended in 100% ethanol, and the absorption of the ethanol solution at 590 nm was measured. The process was repeated for each plate on consecutive days. The 50% growth inhibitory concentration (GI_50_) values were calculated from the percentage of surviving cells after AbE treatments of 0, 0.00057, 0.0017, 0.005, 0.0085, 0.015, 0.026, and 0.045% by a four-parameter logarithmic method using GraphPad Prism 6 (GraphPad Software Inc., La Jolla, CA, USA).

### Flow cytometry

The cells were plated in the appropriate culture medium at a density of 2.5 × 10^5^ cells/plate in 10-cm dishes. After incubation for 24 h, the cells were treated with 0.005, 0.015%, or 0.045% (*w*/*v*) AbE for 48 h. For the control (MOCK), an equal volume of water as AbE supplement was added to the medium. The cells were washed with PBS(−) and fixed overnight with 70% ethanol at − 20 °C. The fixed cells were resuspended in 100 μL hypotonic citrate buffer (192 mmol/L Na_2_HPO_4_, 4 mmol/L citric acid) and incubated for 20 min at room temperature. The cells were then pelleted and resuspended in PI/RNase/PBS (100 μg/mL propidium iodide and 10 μg/mL RNase A in PBS). DNA content was analyzed using the FACSCalibur™ System (BD Biosciences, San Diego, CA, USA).

### Terminal deoxynucleotidyl transferase dUTP nick-end labeling (TUNEL) assay

The cells were plated using the appropriate culture medium at a density of 2.5 × 10^5^ cells/plate in 10 cm dishes. After incubation for 24 h, the cells were treated with 0.045% (*w*/*v*) AbE. For the control, an equal volume of water was added to the medium of the MOCK cells. After incubation for 48 h, the floating and adherent cells were collected and fixed with 4% paraformaldehyde/PBS overnight at 4 °C. The cells were washed with PBS and then spread on MAS-coated glass slides (Matsunami Glass Industry Ltd., Tokyo, Japan). The TUNEL assay was performed to identify DNA fragmentation in situ by using a FragEL™ DNA fragmentation Detection Kit, Colorimetric-TdT Enzyme (Calbiochem, San Diego, CA, USA) in accordance with the manufacturer’s protocol. Approximately 500 cells from randomly selected fields were observed at 400× magnification, from which the number of TUNEL-positive cells was counted.

### Microarray

Total RNA was extracted from cells treated with 0.045% (*w*/*v*) AbE or water for 48 h by using the RNeasy Midi Kit (QIAGEN, Hilden, Germany) in accordance with the supplier’s instructions. The extraction was repeated independently in triplicate. cRNAs labeled with Cyanine 3 were synthesized via the T7-linear amplification method from 500 ng of total RNA using the Low RNA Input Linear Amplification Kit (Agilent Technologies, Palo Alto, CA, USA), and subsequently purified by using the RNeasy Mini Kit (QIAGEN). For each hybridization, 1.65 μg of cRNAs was fragmented at 60 °C for 30 min using the Gene Expression Hybridization Kit (Agilent Technologies) and hybridized at 65 °C for 17 h to a 4 × 44 K Whole Human Genome Microarray slide (Agilent Technologies) in accordance with the manufacturer’s instructions. The microarrays were scanned and the signals in the scanned image were converted to intensity values that were subsequently normalized by software provided by Hokkaido System Science Co., Ltd. (Sapporo, Japan). The expression levels were normalized to the 75th percentile, and baseline transformations were conducted by using the median of the control samples from each cell line. As a means of quality control, the correlation coefficients were analyzed; one sample of PK-8 cells treated with AbE was excluded because its correlation coefficient with the other replicates was below 0.01. Lists of genes with greater than four-fold change in expression were identified by t-test (*P* < 0.05) using R (https://www.r-project.org/). Gene Ontological category lists containing genes with statistically significant expression levels were obtained using Fisher’s exact tests, using Benjamini-Yekutieli corrections (*P* < 0.05). The whole microarray data set was deposited to Gene Expression Omnibus (https://www.ncbi.nlm.nih.gov/geo/) under the accession number GSE89396. Over-representation analyses were performed using online the programs of PANTHER (http://www.pantherdb.org) [26] and REACTOME (https://www.reactome.org) [27].

### Immunoblotting

Pancreatic cancer cells treated with 0.045% (*w*/*v*) AbE or an equal volume of water for 48 h were harvested by mild scraping, centrifuged, and washed with ice-cold PBS(−) containing Complete Mini protease inhibitors (Roche, Basel, Switzerland). The cells were then suspended in an appropriate volume of RIPA buffer (Sigma-Aldrich) plus protease inhibitors (Roche) and lysed by sonication. The lysates (120 mg) were denatured, separated on SDS-polyacrylamide gels (Bio-Rad, Hercules, CA, USA), and then electroblotted onto Clear Blot membrane-p (ATTO, Tokyo, Japan). An SDS gradient gel of 5–20% polyacrylamide was used for poly (ADP-ribose) polymerase 1 (PARP1) and 10–20% polyacrylamide was used for caspase-3, caspase-9, and β-actin. After non-specific binding to the membrane was blocked by incubation with TBST (20 mM Tris-HCl, pH 7.6, 137 mM NaCl, and 0.1% Tween® 20) containing 5% (*w*/*v*) Amersham ECL Blocking Agent (GE Healthcare UK Ltd., Buckinghamshire, UK), the membranes were incubated overnight at 4 °C with Can Get Signal® Solution 1 (TOYOBO, Osaka, Japan) containing the following antibodies, separately: mouse monoclonal anti-PARP1 (clone 42/PARP, dilution 1:125, BD Biosciences, San Jose, CA, USA), mouse monoclonal anti-caspase-3 (clone 3G2, dilution 1:1000, Cell Signaling Technology Inc., Beverly, MA, USA), rabbit polyclonal anti-caspase-9 (dilution 1:1000, Cell Signaling Technology Inc.), or mouse monoclonal anti-β-actin (clone AC-15, dilution 1:1000, Sigma-Aldrich). Then, the membranes were incubated with Can Get Signal® Solution 2 (TOYOBO) containing an appropriate horseradish peroxidase (HRP)-conjugated anti-mouse or anti-rabbit immunoglobulin secondary antibody (dilution 1:10000, GE Healthcare UK Ltd.) at room temperature for 1 h. The bound antibodies were detected by the application of enhanced chemiluminescence (ECL) solution (Amersham ECL Plus™ Western Blotting Detection Reagents, GE Healthcare UK Ltd.) and digitally processed using an LAS-4000 mini image analyzer (Fuji Photo Film Co. Ltd., Minamiashigara, Japan).

### Quantitative reverse transcription PCR

Single-stranded cDNA was synthesized from the total RNA used for the microarray analysis using the High-Capacity cDNA Reverse Transcription Kit (Applied Biosystems, Foster City, CA, USA) in accordance with the manufacturer’s instructions. Quantitative PCR was performed using the 7500 Real-Time PCR System (Applied Biosystems) with the synthesized cDNAs and Pre-Developed TaqMan® Assay Reagents (Applied Biosystems) for glyceraldehyde 3-phosphate dehydrogenase (*GAPDH;* 4326317E) or TaqMan® Gene Expression Assays (Applied Biosystems) for cyclin D1 (*CCND1;* Hs00277039_m1), cyclin-dependent kinase inhibitor 3 (*CDKN3;* Hs00193192_m1), cyclin B2 (*CCNB2;* Hs00270424_m1), cyclin A2 (*CCNA2;* Hs00996789_g1), cyclin F (*CCNF;* Hs00171049_m1), CHK2 checkpoint homolog (*S. pombe*) (*CHEK2;* Hs00200485_m1), ataxia telangiectasia mutated (*ATM;* Hs00175892_m1), cell division cycle 25 homolog A (*S. pombe*) (*CDC25A;* Hs00153168_m1), breast cancer 1, early onset (*BRCA1;* Hs00173237_m1), high mobility group (HMG)-box transcription factor 1 (*HBP1;* Hs00202110_m1), forkhead box O4 (*FOXO4;* Hs00172973_m1), vasohibin 1 (*VASH1;* Hs00208609_m1), BRCA1 associated RING domain 1 (*BRAD1;* Hs00184427_m1), cyclin-dependent kinase 6 (*CDK6;* Hs00608037_m1), NLR family pyrin domain containing 1 (*NLRP1;* Hs00248187_m1), death effector domain containing 2 (DEDD2; Hs00370206_m1), or death-associated protein kinase 3 (*DAPK3;* Hs00154676_m1). The PCR cycling program consisted of 95 °C for 10 min, followed by 40 cycles of 95 °C for 15 s and 60 °C for 1 min. All samples were analyzed in triplicate and gene expression was calculated relative to the reference housekeeping gene, *GAPDH*.

## Results

### AbE attenuates proliferation of human pancreatic cancer cells

**Fig. 1:**
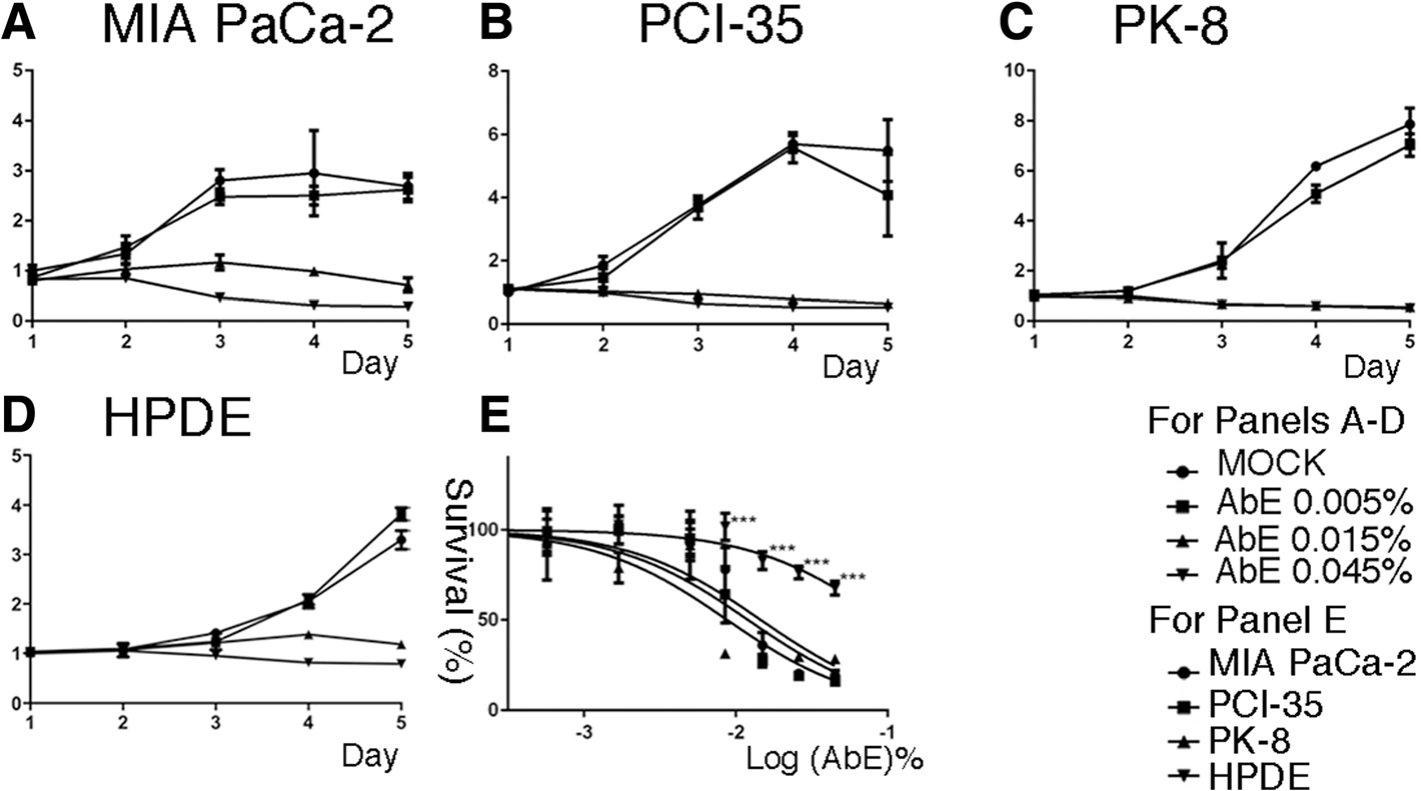
**a-d** Attenuation of the proliferation of human pancreatic cancer cells compared with that of human normal pancreatic duct epithelial (HDPE) cells by hot water extract of *Agaricus blazei* Murrill (AbE). Relative proliferation of pancreatic cancer cell lines (MIA PaCa-2, PCI-35, and PK-8) and HPDE treated with AbE at the indicated concentrations, measured by using the MTT assay. The plots represent the average of eight replicates, and the error bars indicate the standard deviation. Y axis denotes a relative growth rate to the mean value of MOCK on Day 1. **e** Cell death curves of pancreatic cancer cells and HPDE cells treated for 48 h with serial dilutions of AbE. An unpaired t-test with the Holm-Sidak method for multiple comparisons correction was used to compare HPDE to pancreatic cancer cells. ***p < 0.001

First, we explored the effect of AbE on the proliferation of human pancreatic cancer cells of genetically and histologically distinct origins: MIA PaCa-2, PCI-35, and PK-8 [28]. The cells were incubated with appropriate growth medium supplemented with AbE at final concentrations (*w*/*v*) of 0.005, 0.015%, or 0.045%, and cellular proliferation was then monitored for five consecutive days by using the MTT assay. We found that AbE significantly attenuated proliferation of all the pancreatic cancer cell lines in a concentration-dependent manner (Fig. 1a-c). Although the effect of 0.005% AbE was mild, 0.015% AbE was sufficient to induce almost complete blockage of cell proliferation in all three cell lines. To compare the effect of AbE on pancreatic cancer cells with its effect on normal pancreatic ductal cells, we treated an immortalized human pancreatic duct epithelial cell line, HPDE [25], with AbE under the same conditions. The results revealed that HPDE was less sensitive, i.e., more resistant, to AbE than pancreatic cancer cell lines (Fig. 1d, e). The GI_50_ values of AbE treatment for the tested cell lines were 0.015% for MIA PaCa-2, 0.012% for PCI-35, 0.009% for PK-8, and 0.094% for HPDE. These results indicated that AbE significantly and specifically inhibited the proliferation of pancreatic cancer cells relative to that of normal duct-epithelial cells.

### AbE induces apoptotic cell death in pancreatic cancer cells

**Fig. 2:**
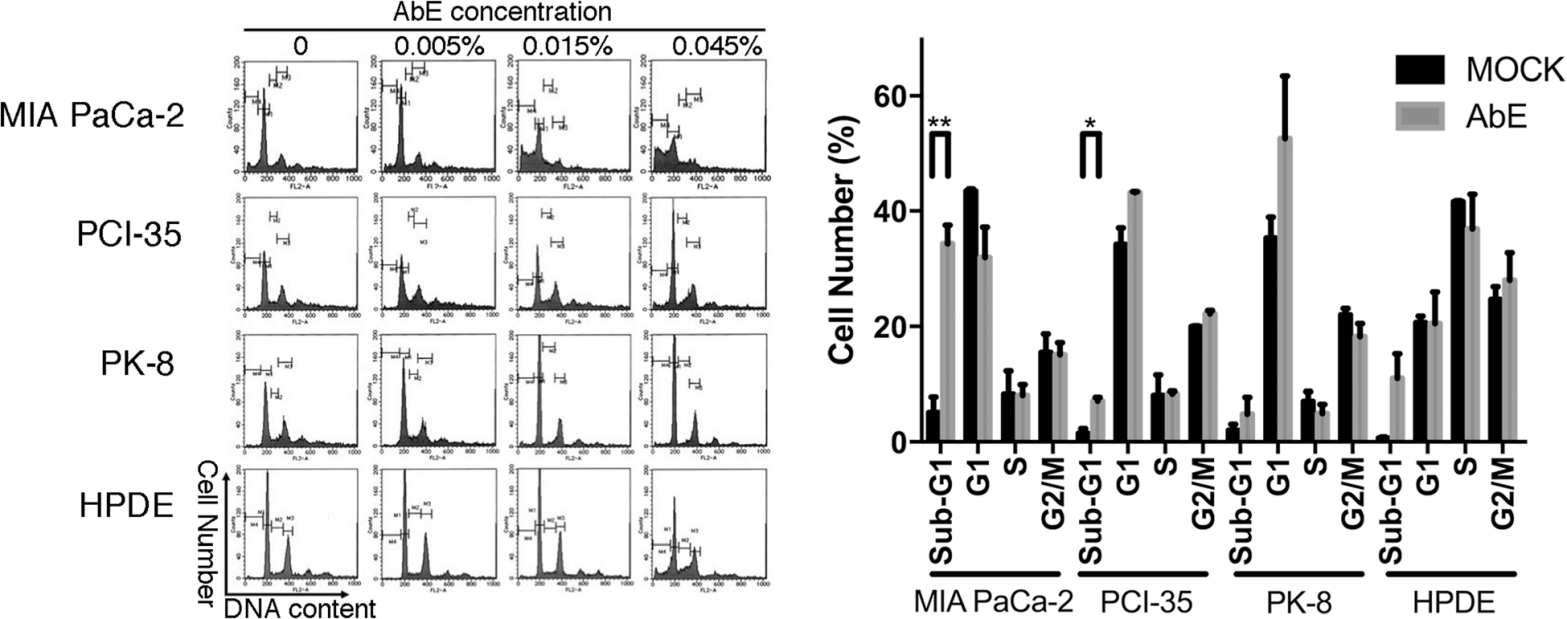
Hot water extract of *Agaricus blazei* Murrill (AbE) induces cell cycle arrest and apoptosis in pancreatic cancer cells. A. Flow cytometric analysis of the cell cycle phase fractions of pancreatic cancer cells (MIA PaCa-2, PCI-35, and PK-8) and the human normal pancreatic duct epithelial (HDPE) cells treated with AbE at the indicated concentrations for 48 h. B. A plot of the relative number of cells in cell cycle fractions including sub-G1 fraction after 0.045% AbE treatment, obtained using flow cytometric analysis

**Fig. 3:**
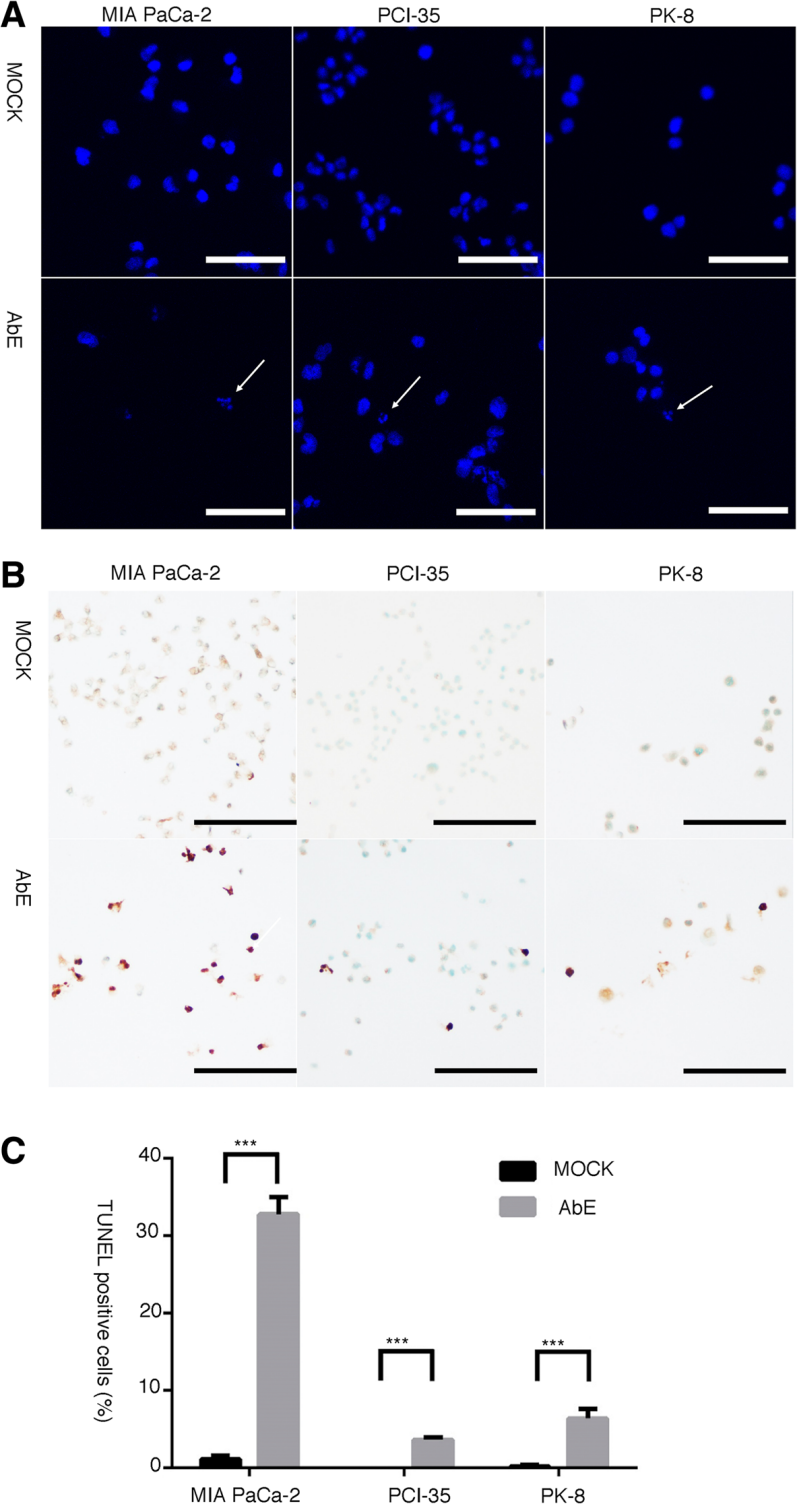
Pancreatic cancer cells treated with hot water extract of *Agaricus blazei* Murrill (AbE) undergo apoptotic cell death. **a** DAPI staining indicates fragmented nuclei in AbE-treated cells (arrows). **b** In situ terminal deoxynucleotidyl transferase-mediated dUTP nick-end labeling (TUNEL) assay indicating DNA fragmentation in AbE-treated cells. **c** The number of TUNEL-positive cells increased with AbE treatment. ***p < 0.001

To elucidate the mechanism of the inhibition of cell proliferation, we used flow cytometry to quantify the cell cycle phase distribution and the sub-G1 DNA content of pancreatic cancer cells treated with AbE for 48 h. The treatment significantly increased the proportion of cells in the G1 or G2 fraction, with a variable increase in the population of the sub-G1 fraction, which indicated that AbE induced G1 or G2 arrest and apoptosis in pancreatic cancer cells (Fig. 2). Morphological examination of nuclei stained with DAPI (4′,6-diamidino-2-phenylindole) revealed increased nuclear fragmentation in AbE-treated cells compared with control-treated cells (Fig. 3). TUNEL assays demonstrated that the number of TUNEL-positive cells actually increased after AbE treatment (Fig. 3). These results provided further evidence that AbE induces apoptotic cell death of pancreatic cancer cells.

**Fig. 4:**
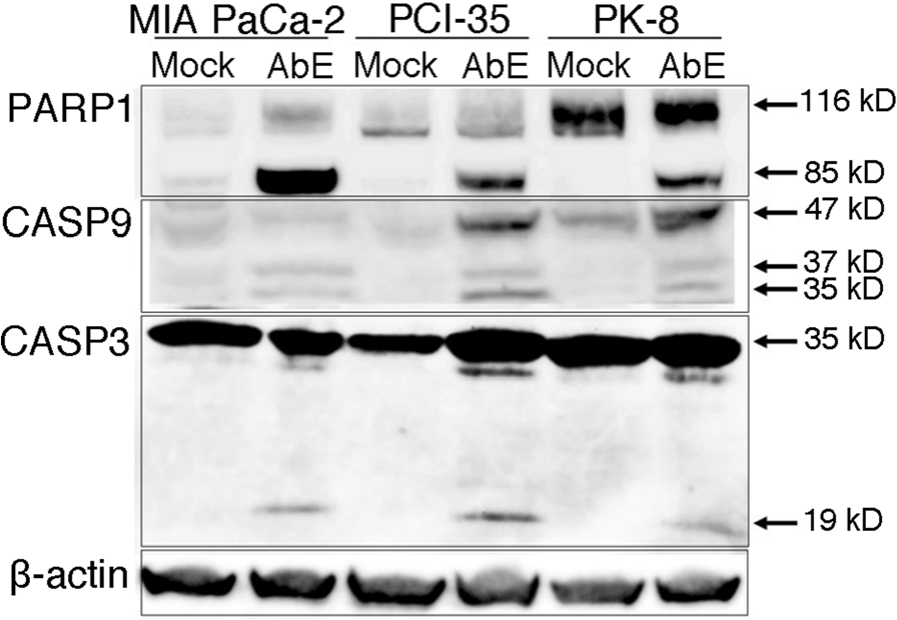
Hot water extract of *Agaricus blazei* Murrill (AbE) induces caspase-dependent apoptosis. Immunoblots indicate the expression of caspase-3 (CASP3), caspase-9 (CASP9), poly (ADP-ribose) polymerase (PARP), and their active cleaved products. Immunostaining for β-actin was used as the loading control

To determine whether the AbE-induced apoptosis was caspase-dependent, the cleavage of caspase-3, caspase-9, and PARP1 was examined: AbE treatment induced cleavage of these molecules, which indicated that AbE induces apoptosis through the caspase-dependent pathway (Fig. 4).

### Alteration of global gene expression by AbE treatment in pancreatic cancer cells

**Fig. 5:**
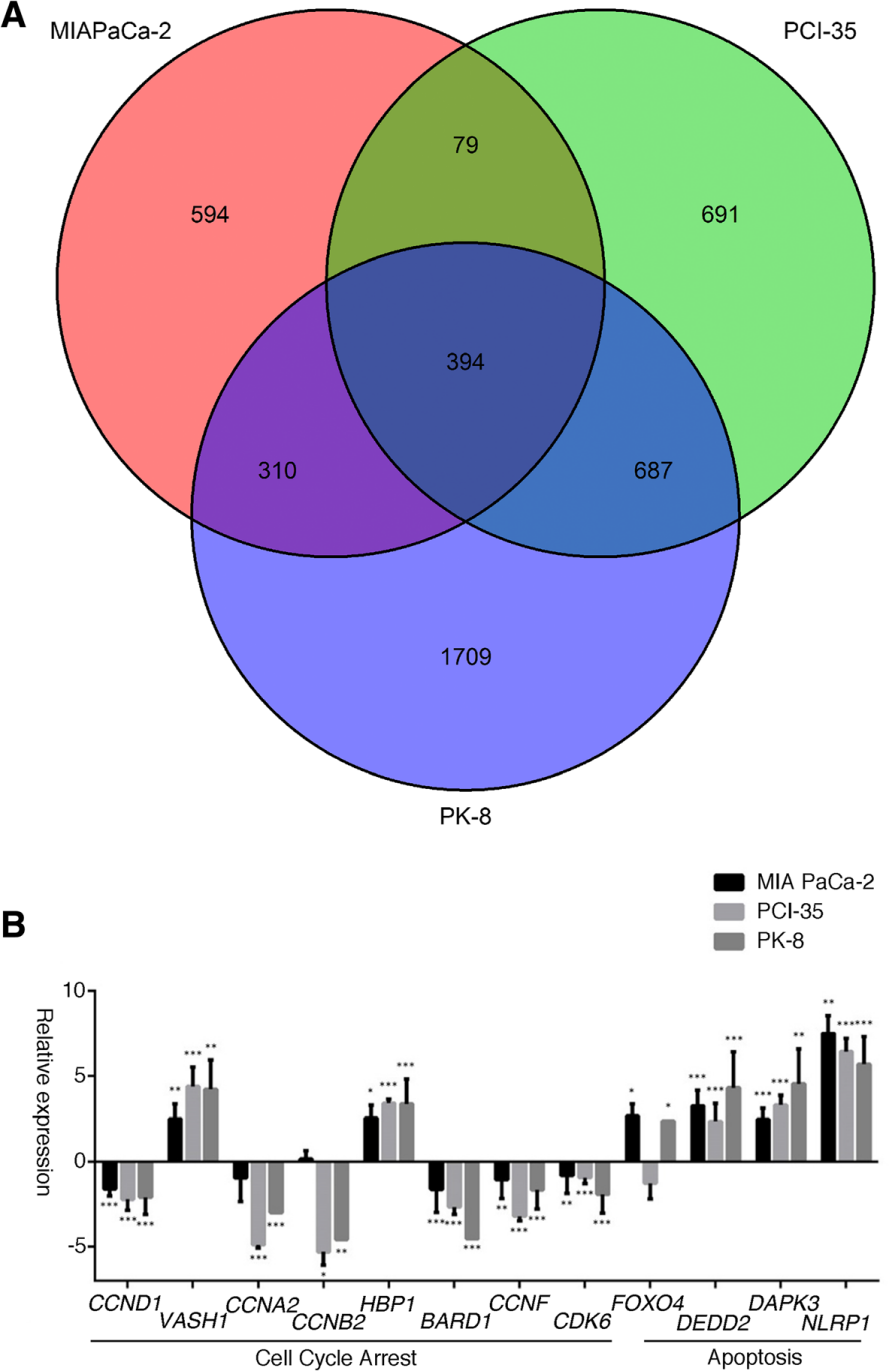
**a** Venn diagram analysis of global gene expression of pancreatic cancer cells treated with the hot water extract of *Agaricus blazei* Murrill (AbE). Genes with a statistically significant (greater than four-fold) change in expression were collected and classified. In the three cell lines, 394 genes were commonly altered. **b** Quantitative reverse transcription PCR was performed to validate the microarray results. The expression of genes associated with cell cycle progression or apoptosis was altered by the AbE treatment. The mean changes in gene expression relative to those in untreated cells, calculated by using *GAPDH* as the reference housekeeping gene, are displayed on a log2 scale. The difference in expression of each mRNA relative to that of non-treated cells was determined using a Student’s t-test. *p < 0.05, **p < 0.01, ***p < 0.001

To determine the effect of AbE on gene expression in human pancreatic cancer cells, global gene-expression profiles were investigated using a whole human genome microarray. The data of the microarray results were deposited at the Gene Expression Omnibus (https://www.ncbi.nlm.nih.gov/geo/) under the accession number GSE89396. We focused on genes with at least a statistical significantly four-fold change in expression following AbE treatment, and then performed Venn diagram analysis. Of the examined genes, 394 were commonly altered in all pancreatic cancer cells, of which 210 were downregulated and 184 were upregulated (Fig. 5a, Additional file 1: Table S1). The PANTHER statistical over-representation test based on the Gene Ontology (GO) database (http://www.pantherdb.org/; released 2016-09-24) was conducted to investigate the functional aspects of the commonly altered genes (Additional file 2: Table S2). In the analysis using the GO biological process database, the genes significantly overrepresented were associated with the kinetochore, spindle checkpoint, mitotic prometaphase, centromere protein A (CENP-A)-containing nucleosome assembly, mitotic cytokinesis, G2 DNA damage checkpoint, sister chromatid cohesion, regulation of mitotic metaphase/anaphase transition, mitotic sister chromatid segregation, regulation of the G2/M transition of mitotic cell cycle, mitotic anaphase, establishment of chromosome localization, mitotic cell cycle checkpoint, negative regulation of mitotic cell cycle phase transition, and positive regulation of cell cycle process (Additional file 3: Table S3). In the analysis using the GO molecular function database, genes associated with protein binding were significantly overrepresented (Additional file 3: Table S3). In the analysis using the GO cellular component database, genes associated with Ndc80 complex, spindle midzone, midbody, and spindle pole were significantly overrepresented (Additional file 3: Table S3). We also performed another over-representation test using REACTOME and confirmed that genes associated with the mitotic spindle checkpoint were significantly overrepresented (*p* = 3.19E-8; Additional file 4: Table S4). We performed an additional analysis to see if any genes were specifically overrepresented in a particular cell line, but we did not find any such genes.

Then, we analyzed genes specifically altered in each cell line by AbE treatment because each cell responded differently; in detail, MIA PaCa-2 showed marked G2 arrest and apoptosis; PCI 35 showed G1 arrest and apoptosis; and PK-8 showed less significant G1 arrest and apoptosis (Fig. 2). As shown in Fig. 5, 594, 691, and 1709 genes were specifically altered 4-fold in MIA PaCa-2, PCI-35, and PK-8, respectively. Among them, 297, 361, and 888 genes were annotated by GO Biological Process in MIA PaCa-2, PCI35, and PK-8, respectively. Each had unique genes associated with cell cycle, cell cycle arrest, G1 phase, G2 phase, and apoptosis (Additional file 5: Table S5).

We validated the alterations in expression of genes associated with cell cycle, namely *CCND1, VASH1, CCNA2, CCNB2, HBP1, BARD1, CCNF, CDK6,* and *FOXO4,* and those associated with apoptosis, namely *DEDD2, DAPK3,* and *NLRP1*, by using the quantitative reverse transcription PCR [29–34]. The validation confirmed the downregulation of cell cycle-promoting genes (*CCND1, CCNA2, CCNB2, BARD1, CCNF,* and *CDK6*) and the upregulation of cell cycle arrest genes (*VASH1* and *HBP1*) and proapoptotic genes (*DEDD2, DAPK3*, and *NLRP1*) in all cell lines tested in this study (Fig. 5b).

These results of global gene expression analyses indicated that AbE treatment impaired kinetochore function, spindle formation, centromere maintenance, cyclins, and cyclin-dependent kinases, which are essential for cell cycle progression.

## Discussion

In this study, we revealed that AbE treatment attenuated the proliferation and induced caspase-dependent apoptotic cell death of human pancreatic cancer cells. The attenuation of cell proliferation was less remarkable in cells from the normal pancreatic duct. Moreover, we found that AbE treatment drastically altered the expression of genes mainly associated with kinetochore function, mitosis, and cell cycle progression. These results indicated that AbE may be useful for the treatment of pancreatic cancer and serve as a candidate for isolation of novel chemical compounds that specifically alter spindle function.

From our analysis of the effect of AbE on the global gene expression of human pancreatic cancer cells, AbE seems to repress genes specifically associated with kinetochore function, spindle formation, centromere maintenance, and regulation of cyclins. *CASC5* encodes the protein, kinetochore scaffold 1, which is a component of the multiprotein assembly required for the formation of kinetochore-microtubule attachments and chromosome segregation. *BUB1* and *BUB1B* encode the proteins, BUB1 mitotic checkpoint serine/threonine kinase and BUB1 mitotic checkpoint serine/threonine kinase B, respectively; these function by phosphorylating the members of the mitotic checkpoint complex and activating the spindle checkpoint. *PLK1* encodes the protein, polo like kinase 1, which participates in DNA replication and repair, cell cycle progression, centriole duplication, and centrosome maturation [35]. *AURKB* encodes the protein, aurora kinase B, which participates in the regulation of alignment and segregation of chromosomes during mitosis and meiosis through association with microtubules. *KNTC2* encodes a component of the Ndc80 kinetochore complex that organizes and stabilizes microtubule-kinetochore interactions and is required for proper chromosome segregation. *ZWINT* encodes the ZW10 interacting kinetochore protein that is possibly involved in kinetochore function via the regulation of the association between ZW10 and kinetochores. *CENPA*, *CENPE*, *CENPF*, *CENPI*, *CENPU/MLF1IP*, and *CENPW/C6orf173* encode centromere proteins that are vital for centromere functions. *CCNA2*, *CCNB2*, and *CCND1* encode cyclins, and *CDC2/CDK1*, *CDC7*, *CDC20*, and *CDCA2* encode cell division cycle proteins that are required for progression of the cell cycle. *HBP1* encodes the HMG-box transcriptional factor 1 that represses the function of CCND1 [36]. *CIT* encodes the citron rho-interacting serine/threonine kinase that is active in cell division.

Apoptosis is a biological process that eliminates redundant, damaged, or infected cells [37]; apoptotic pathways are disrupted in many cancers. Moreover, these disrupted apoptotic pathways are associated with the chemoresistance of pancreatic cancer [38]. Our analyses of the pancreatic cancer cells treated with AbE consistently showed that they led to apoptotic cell death that depended on caspase activation. Caspases are critical components of apoptosis and are activated by proteolytic cleavage. Caspase-3 is one of the central effector caspases for both the intrinsic and extrinsic apoptotic pathways. Caspase-3 is activated by the caspase-9 apoptosome in the intrinsic pathway, and it cleaves PARP1 into 24 kDa and 89 kDa fragments, leading to inhibition of DNA repair enzymes. We also showed that the genes encoding proapoptotic proteins, namely DEDD2, DAPK3, and NLRP1, were overexpressed in pancreatic cancer cell lines treated with AbE. Overexpression of DEDD2 can induce apoptosis by binding and activating caspase-8/caspase-10, and sensitizing cells to apoptosis triggered by CD95 or tumor necrosis factor-related apoptosis-inducing ligand (TRAIL) [29, 30]. DAPK3 is a p53-activating kinase and overexpression of DAPK3 can lead to apoptosis [31, 32]. DAPK3 is required for caspase activation together with death-domain associated protein (DAXX) [33]. NLRP1 overexpression can also induce apoptosis in breast adenocarcinoma cells [34]. Among the genes repressed by AbE treatment, *GTSE1* encodes the protein G2 and S-phase expressed 1 (GTSE1), which is only expressed in the S and G2 phases of the cell cycle, where it colocalizes with cytoplasmic tubulin and microtubules. It has been reported that GTSE1 accumulates in the nucleus in response to DNA damage and binds p53, shuttling the latter out of the nucleus and suppressing its ability to induce apoptosis [39]. Given that the p53 pathway commonly malfunctions in pancreatic cancer, and that p53 is often disrupted in pancreatic cancer cells by mutation or loss of expression [28], it is remarkable that AbE can induce apoptotic cell death in such cells.

In contrast, the cells responded differently to AbE. MIA PaCa-2 showed G2 arrest and apoptosis, whereas PCI-35 and PK-8 showed mainly G1 arrest. We analyzed genes potentially associated with these cell-specific characteristic features, which included many genes associated with cell cycle arrest and apoptosis (Additional file 5: Table S5).

The isolation of active compounds from natural products is a classic and powerful method for the discovery of new efficacious treatments for disease. Several compounds have been isolated from AbE that showed antitumor activity [9, 40–42]: polysaccharides, agaritine, and a phenylhydrazine containing a glutamyl residue. The compounds responsible for the impairment of mitotic spindle function that can induce apoptotic cell death in pancreatic cancer cells should be identified.

## Conclusion

The results of this study indicate that the hot water extract of *A. blazei* could be useful for the treatment of pancreatic cancer and is a potential candidate for the isolation of novel active compounds to impair mitotic spindle function.

## Additional files

Additional file 1: **Table S1.** Genes with a statistically significant (greater than four-fold) change in expression in human pancreatic cancer cells treated with hot water extract of *Agaricus blazei* Murrill relative to the gene expression induced by mock treatment. (XLS 28046 kb)

Additional file 2: **Tables S2**. PANTHER Overrepresentation Test based on Gene Ontology (GO) data. (XLS 40 kb)

Additional file 3: **Tables S3**. Genes significantly overrepresented by PANTHER Overrepresentation Test based on Gene Ontology (GO) data. (XLS 108 kb)

Additional file 4: **Table S4**–1. Overrepresentation test using REACTOME. **Table S4**–2. Genes overrepresented by REACTOME analysis. (XLS 31 kb)

Additional file 5: **Table S5**–1. GO Biological Process annotations for genes altered only in MIA PaCa-2 by AbE treatment. **Table S5**–2. GO Biological Process annotations for genes altered only in PCI-35 by AbE treatment. **Table S5**–3. GO Biological Process annotations for genes altered only in PK-8 by AbE treatment. (XLS 996 kb)

## Abbreviations

5-fluorouracil: Irinotecan, and oxaliplatin
AbE: A hot water extract of *A. blazei*
CAM: Complementary and alternative medicine
DAPI: 4′,6-diamidino-2-phenylindole
ECL: Enhanced chemiluminescence
FOLFIRINOX: Folinic acid
GI_50_: The 50% growth inhibitory concentration
HRP: Horseradish peroxidase
MTT: 3-(4,5-dimethylthiazol-2-yl)-2,5-diphenyltetrazolium bromide
PARP1: Poly (ADP-ribose) polymerase 1
TNF-α: Tumor necrosis factor-α
TUNEL: The terminal deoxynucleotidyl transferase dUTP nick-end labeling assay
